# Assessing the accuracy of the recording and reporting of malaria rapid diagnostic test results in four African countries: methods and key results

**DOI:** 10.1186/s12936-025-05459-7

**Published:** 2025-07-01

**Authors:** Kim A. Lindblade, Arthur Mpimbaza, Corine Ngufor, William Yavo, Sunday Atobatele, Ese Akpiroroh, Abibatou Konaté-Touré, Idelphonse Ahogni, Nelson Ssewante, Bosco Agaba, Augustin Kpemasse, Jacques Agnon, Onyebuchi Okoro, Godwin Ntadom, Antoine Tanoh, Cyriaque Affoukou, Jimmy Opigo, Shawna Cooper, John J. Aponte, Kevin Griffith, Radina Soebiyanto, Michael Humes

**Affiliations:** 1 PMI Insights Project/PATH, Rue du Varembé 7, 1202 Geneva, Switzerland; 2 Child Health and Development Centre, Makerere University, Kampala, Uganda; 3 Centre de Recherche Entomologique de Cotonou, Cotonou, Benin; 4 Institut National de Santé Publique, Abidjan, Côte d’Ivoire; 5 Sydani Group, Abuja, Nigeria; 6 National Malaria Control Division, Kampala, Uganda; 7 Programme National de Lutte Contre le Paludisme, Cotonou, Benin; 8 Programme National de Lutte Contre le Paludisme, Abidjan, Côte d’Ivoire; 9 National Malaria Elimination Programme, Abuja, Nigeria; 10 Audere, Seattle, WA USA; 11 U.S. President’s Malaria Initiative, Washington, DC USA

**Keywords:** Malaria, Rapid diagnostic tests, Diagnosis, Health management information systems, Surveillance, Data quality, Case management, Sub-Saharan Africa

## Abstract

**Background:**

Rapid diagnostic test (RDT) results are the foundation of both case management and malaria surveillance across sub-Saharan Africa. However, RDT results may be misrecorded in health facility registers by healthcare workers (HCWs), either unintentionally or deliberately, for example, to justify treatment based on clinical judgment. A multi-country evaluation was conducted to quantify the extent of RDT misrecording and identify factors associated with recording and reporting accuracy. This report summarizes the study’s methods, key findings, and implications for improving malaria RDT data quality.

**Methods:**

A mixed-methods observational study was implemented in Benin, Côte d'Ivoire, Nigeria, and Uganda in 2023. At participating health facilities, images of RDTs were captured at the point of care and later reviewed by a trained, external panel. Agreement between the panel’s interpretation and the result recorded in the register was assessed using weighted mean Cohen’s kappa (κ). Secondary objectives included identifying factors associated with inaccurate recording, assessing the durability of RDT results after 1 month of storage, evaluating accuracy of data obtained from the District Health Information System 2 (DHIS2), and measuring the observer effect of the start of the study on test positivity rates (TPR) reported to DHIS2.

**Results:**

A total of 102,337 RDT results was observed. Agreement between register-recorded results and the external panel was high, ranging from κ = 0.80 (95% confidence interval [CI] 0.75, 0.85) in Nigeria to κ = 0.88 (95% CI 0.84, 0.92) in Benin. HCWs were more likely to misrecord results as positive (range: 5.1–7.3%) than negative (range: 0.7–3.7%), and patient age was associated with misrecording results in all countries except Nigeria. After the study began in Côte d’Ivoire, TPRs reported from the DHIS2 declined significantly more in study than control facilities (rate ratio: 0.80, 95% CI 0.76, 0.84).

**Conclusions:**

Although HCWs generally recorded RDT results accurately, the disproportionately higher proportion of results misrecorded as positive raises concern about possible intentional misreporting. The observed TPR decrease after study onset in Côte d’Ivoire suggests some HCWs can be motivated to reduce misrecording and improve the quality of malaria case management and surveillance data.

**Supplementary Information:**

The online version contains supplementary material available at 10.1186/s12936-025-05459-7.

## Background

The foundation of both case management and surveillance for malaria is the diagnostic confirmation of infection in persons with suspected malaria. The development of malaria rapid diagnostic tests (RDTs), which are small cassettes containing immunochromatographic strips that detect malaria-specific antigens in blood obtained from a finger-prick, has revolutionized testing for malaria. From 2010 to 2023, the annual number of suspected malaria cases across sub-Saharan Africa (SSA) receiving a diagnostic test increased more than five-fold from 54 to 334 million [1]. This increase was almost entirely driven by the scale-up of malaria RDTs, which rose from 20 million instances in 2010 to 266 million in 2023. As a result, malaria treatment specificity has improved and routine surveillance data now rely primarily on parasitological confirmation rather than presumptive diagnosis.

The World Health Organization (WHO) recommends that all patients with suspected malaria undergo parasitological confirmation with either microscopy or RDT before starting antimalarial treatment. However, presumptive treatment may be initiated if diagnostics are not immediately available [2]. Patients with confirmed malaria should receive a full course of antimalarial treatment while those who test negative should not be given antimalarials. Instead, further evaluation is needed to identify the cause of illness and guide appropriate treatment. In SSA, however, prescription of antimalarials to those who test negative for malaria is widespread: a systematic review found that 22% (95% confidence interval [CI] 11, 34) of RDT-negative patients were still treated with antimalarials by healthcare workers (HCWs) [3].

While diagnostic tests are essential tools in clinical decision-making, they do not, on their own, determine a clinical diagnosis. HCWs may choose to rely on clinical judgment over RDT results due to known limitations of the tests: depending on the specific product and country context, RDTs can miss low density infections [4]; may fail to detect parasites lacking the histidine-rich protein 2 (HRP2) gene [5]; can remain positive for 2 weeks or longer following treatment [6]; and may not be designed to detect non-*Plasmodium falciparum* species that could be responsible for illness [7]. Beyond these technical limitations of RDTs, treatment decisions may also be shaped by external factors. Public demand for medication can pressure HCWs to prescribe antimalarials irrespective of test results, and the frequent lack of diagnostic tools and appropriate treatments for other febrile illnesses may further contribute to HCWs’ treatment of RDT-negative patients with antimalarials [8, 9].

Several initiatives aimed at reducing presumptive treatment, promoting adherence to RDT results, and strengthening antimalarial supply chain integrity have placed considerable emphasis on aligning the number of positive malaria test results with the number of antimalarial treatments prescribed. The WHO Test, Treat & Track initiative that launched in 2012 emphasized the importance of testing before treating. In alignment with this approach, ministries of health (MOH) routinely assess the frequency of presumptive treatment and HCW adherence to negative test results through supportive supervision and end-user verification surveys [10–12]. Similarly, the US President’s Malaria Initiative recommends monitoring the rational use of antimalarial treatments by routinely comparing the number of test results to numbers of treatments administered [13].

An unintended consequence of these initiatives may have been the creation of incentives for HCWs to intentionally misrecord RDT results in health facility registers to account for the treatments provided. As a result, the reported number of confirmed malaria cases and the test positivity rate (TPR) may be artificially inflated. This misreporting can bias estimates of malaria burden and potentially lead to misguided programmatic or policy decisions.

Although multiple studies have found suboptimal alignment between RDT results, diagnoses and prescribed treatment, most evaluations have relied on patient reinterviews where adherence is assessed based on the result of a second RDT performed by trained research staff [14–16]. These studies typically do not include an objective review of the original RDT cassette interpreted by the HCW at the point of care and are often limited by small sample sizes given the resources required to reinterview a large number of patients. To more accurately assess the extent to which RDT results recorded in health facility registers reflect an objective interpretation of the test outcome, the Malaria RDT Capture and Reporting Assessment (MaCRA) was implemented across public primary health care facilities in Benin, Côte d’Ivoire, Nigeria and Uganda. This paper outlines the common methodology used in the MaCRA study, summarizes key results and discusses their potential implications for malaria surveillance and case management in SSA.

## Methods

While this publication reports on the methods and key findings of the MaCRA study, more detailed statistical methods and complete results will be presented in future publications.

### Study objectives

The primary objective of this study was to assess the accuracy of malaria RDT results recorded in public health facility outpatient and antenatal care registers and reported to the District Health Information System 2 (DHIS2, University of Oslo, Oslo, Norway) platform of the national health management information systems (HMIS) in Benin, Côte d'Ivoire, Nigeria, and Uganda. The study harmonized site selection, data collection procedures, and analysis across countries to enable comparisons while accommodating differences in health systems and national priorities.

The study explored several secondary objectives including: identification of characteristics of health facilities, HCWs, and patients associated with misrecording RDT results; measurement of the stability of RDT cassette results over time; comparison of RDT results reported from the DHIS2 with monthly summary forms and health facility registers; and measurement of the observer effect on TPRs reported from the DHIS2.

### Study design

A mixed-methods, prospective observational study was conducted in selected health facilities across Benin, Côte d'Ivoire, Nigeria, and Uganda in 2023. At baseline, health facility surveys were conducted and assessments were undertaken to evaluate HCWs’ characteristics, knowledge, attitudes, perceptions, behaviours (KAPB), and skill in performing RDTs. Trained research assistants used a digital, artificial intelligence (AI)-powered RDT reader (HealthPulse, Audere, Seattle, WA USA), customized for this study and deployed on smartphones, to photograph all RDTs performed during facility opening hours over a period of 4–6 months. For each RDT, research assistants recorded a unique identifier for the HCWs who performed the RDT or recorded the result along with basic patient demographics, the RDT result recorded in the register, the clinical diagnosis, and the treatment prescribed. All RDT photographs were sent to a trained, external panel for interpretation, and these results were compared to those recorded by HCWs in health facility registers. In Benin, Nigeria and Uganda, research assistants stored RDTs after use and re-photographed them at 1 week and 1 month after initial performance to assess the stability of results over time. Research assistants aggregated RDT results daily from health facility registers and digitized monthly summary forms (MSF) used for entry into the DHIS2. These data were compared with data downloaded from the national DHIS2 after the study concluded to assess reporting accuracy. Changes in TPR reported in DHIS2 following the start of the study were compared with pre-study trends in both study facilities and matched control facilities to evaluate whether the study’s implementation influenced the data reported to the DHIS2.

### Study sites

**Fig. 1:**
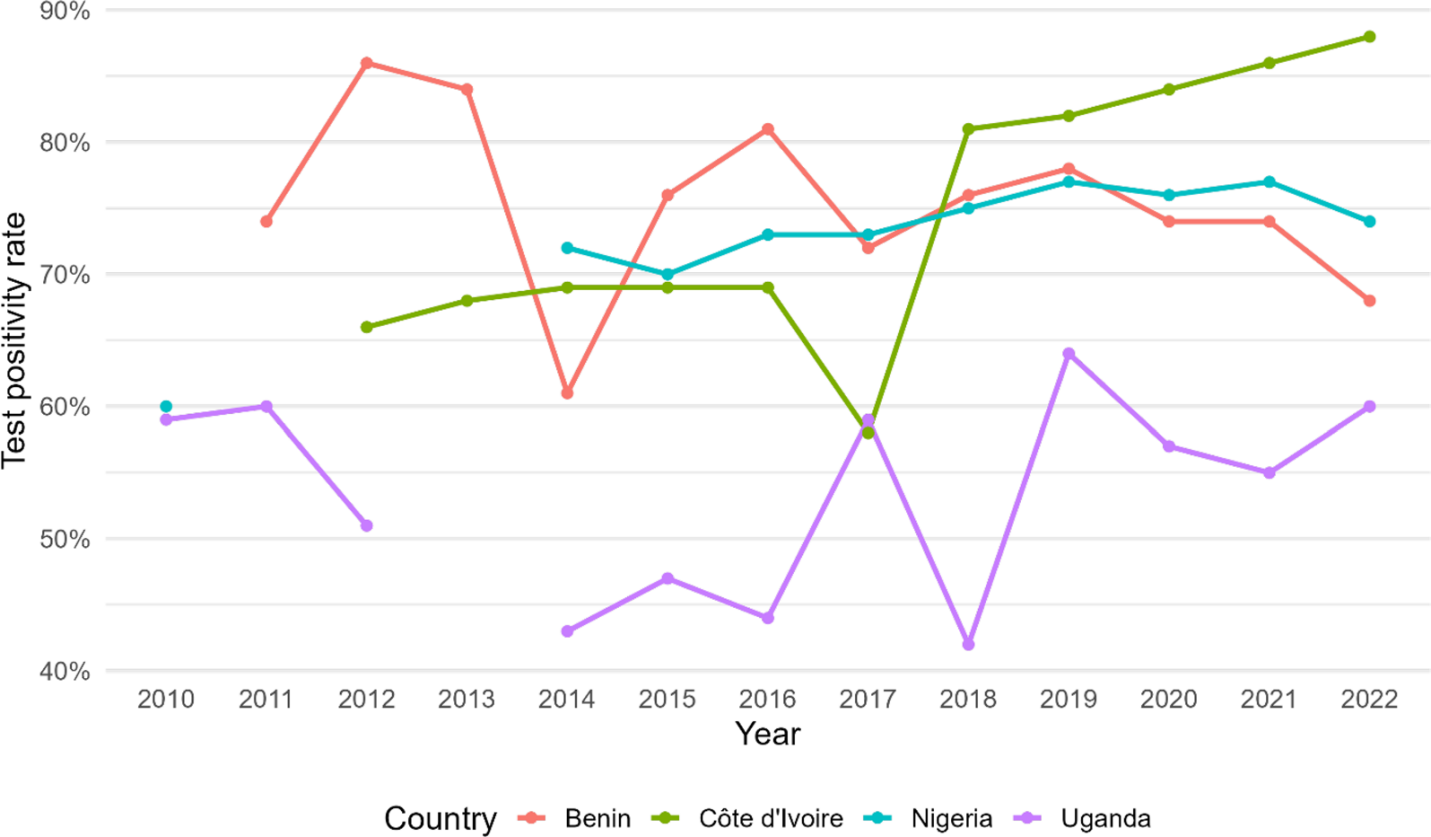
National test positivity rates from rapid diagnostic tests in the four study countries, 2010–2022 [17]

The countries selected for this evaluation were chosen purposively to reflect a range in baseline TPRs in countries supported by the US President’s Malaria Initiative (PMI) (Fig. 1). In all four countries, malaria is a leading cause of morbidity and mortality in children: malaria incidence in 2022 ranged from 262 cases per 1000 population in Côte d’Ivoire to 383 per 1000 population in Benin while malaria mortality per 100,000 population ranged from 40 in Côte d’Ivoire to 87 in Nigeria [17]. In 2022, Côte d’Ivoire tested 50.7% of its suspected malaria cases by RDT, while Benin, Uganda and Nigeria used RDTs to test 79.3%, 81.4% and 84.5%, respectively, of their malaria cases.

Two regions in each country (i.e. departments [Benin], health regions [Côte d’Ivoire], states [Nigeria] or subregions [Uganda]) were purposively selected for the evaluation through discussions with the ministry of health (MOH) and PMI. Benin, Côte d’Ivoire and Nigeria restricted selection to the areas supported by PMI to ensure availability of RDTs and antimalarial medicines during the study and excluded any regions experiencing violence or insurgency, while in Uganda, no geographic restriction was applied as PMI only supported commodities for private facilities. For Benin, Côte d’Ivoire and Nigeria, one region in the north with seasonal transmission of malaria and one region in the south with more perennial transmission were selected. In Uganda, two high-burden subregions were chosen. Within each region, two sub-regional areas (i.e. health zones [Benin], health district [Côte d’Ivoire], local governmental area [Nigeria] or districts [Uganda]) were purposively selected for the evaluation. Benin conducted a further selection of communes within health zones.

Only basic public health facilities were included in this evaluation. In Benin, Côte d’Ivoire and Nigeria, only health facilities without trained laboratorians were eligible for inclusion while in Uganda, facilities with microscopy services were also eligible. No hospitals or referral facilities were included in the evaluation. Public health facilities were further eligible if they reported a minimum average of 50 RDTs per month and had reported data on RDTs to the DHIS2 for at least 9 months out of 12 for each year over the previous 2 years. Health facilities were stratified using medians by patient volume (based on the number of outpatient visits) and average TPR for 2021–2022. Within each of the four strata in each sub-region, one health facility was randomly selected as a study facility resulting in a total of four study facilities per stratum and 16 study facilities per country. One additional health facility was selected from each stratum in each sub-region to serve as a control to measure the observer impact on TPRs reported from the DHIS2; no activities were undertaken and there was no engagement with staff in control health facilities.

### Data collection instruments

Research assistants used smartphones for data collection. Baseline surveys, daily summaries of the health facility register and transcription of the MSF were collected using KoboToolbox (Kobo, Cambridge, MA USA) whereas RDT images and matching patient information from health facility registers were collected using the HealthPulse application. All data collection instruments were implemented in English in Nigeria and Uganda and translated into French for Benin and Côte d’Ivoire using DeepL (Cologne, Germany). French translations were verified by the principal investigators in Benin and Côte d’Ivoire. Data collection tools were piloted in non-evaluation areas and subsequently refined to improve the clarity and flow of the questions.

#### Development and use of the HealthPulse application

The HealthPulse digital RDT reader application was originally developed as a smartphone tool for HCWs to improve performance and interpretation of RDTs. The application has an image quality assurance (IQA) component that leverages computer vision and machine learning processes to assess the quality of images of RDTs, immediately flagging those that do not meet quality standards (such as those with blur or multiple RDTs) and prompting the user to retake the photo. The HealthPulse application includes an AI algorithm that interprets the RDT result from the image. The AI result was not provided to the research assistants, HCWs, external panel or study investigators (except for SC) during the study period and was not used to inform the clinical management of the patient.

Eight RDTs products expected to be used in one or more of the four evaluation countries were identified prior to the start of the study by the MOH; one additional product was added during the course of the study (Supplementary Table 1). All RDT products detected *P. falciparum* using the *P. falciparum*-specific histidine-rich protein 2 (HRP2) antigen. One product had a second test line with a biomarker to detect *P. falciparum*-specific lactate dehydrogenase (*Pf*LDH) and two products had a second test line that detected pan-*Plasmodium* lactate dehydrogenase (pLDH).

The RDT products identified by the MOH were used to develop the HealthPulse AI algorithm. Sets of RDT images that were generated through laboratory production, synthetic imagery and collection of RDT cassettes in health facilities in South Africa were used to train the AI algorithm to interpret results in diverse conditions such as varying lighting, focal lengths and RDT misadministration. The HealthPulse AI algorithm was further trained on RDT images collected during the first 2 weeks of the study in each country; images and data from this period were not included in the analytical dataset.

The HealthPulse application had identical functionality offline as it did online. RDT images and patient data were automatically uploaded to a secure server once an internet connection became available. In Uganda, research assistants working in high-volume facilities with limited connectivity addressed upload backlogs by enabling mobile hotspots or relocating devices to areas with better signal coverage.

### Study procedures

Research assistants in each country underwent a 3–4 day training that included an orientation to the study objectives and research ethics, a review of standardized operating procedures for data collection, and guidance on obtaining informed consent from participants. They were also trained to administer RDTs in order to accurately observe and document how health care workers performed the tests. Research assistants were present during the main working hours of each facility and were instructed to not interact directly with patients and to minimize their impact on routine facility operations. Most countries deployed one research assistant per facility; however, Benin assigned two per facility, as most sites included both outpatient and maternal health clinics. In Nigeria, facilities operating 24 h a day were supported by two shifts of research assistants.

**Fig. 2:**
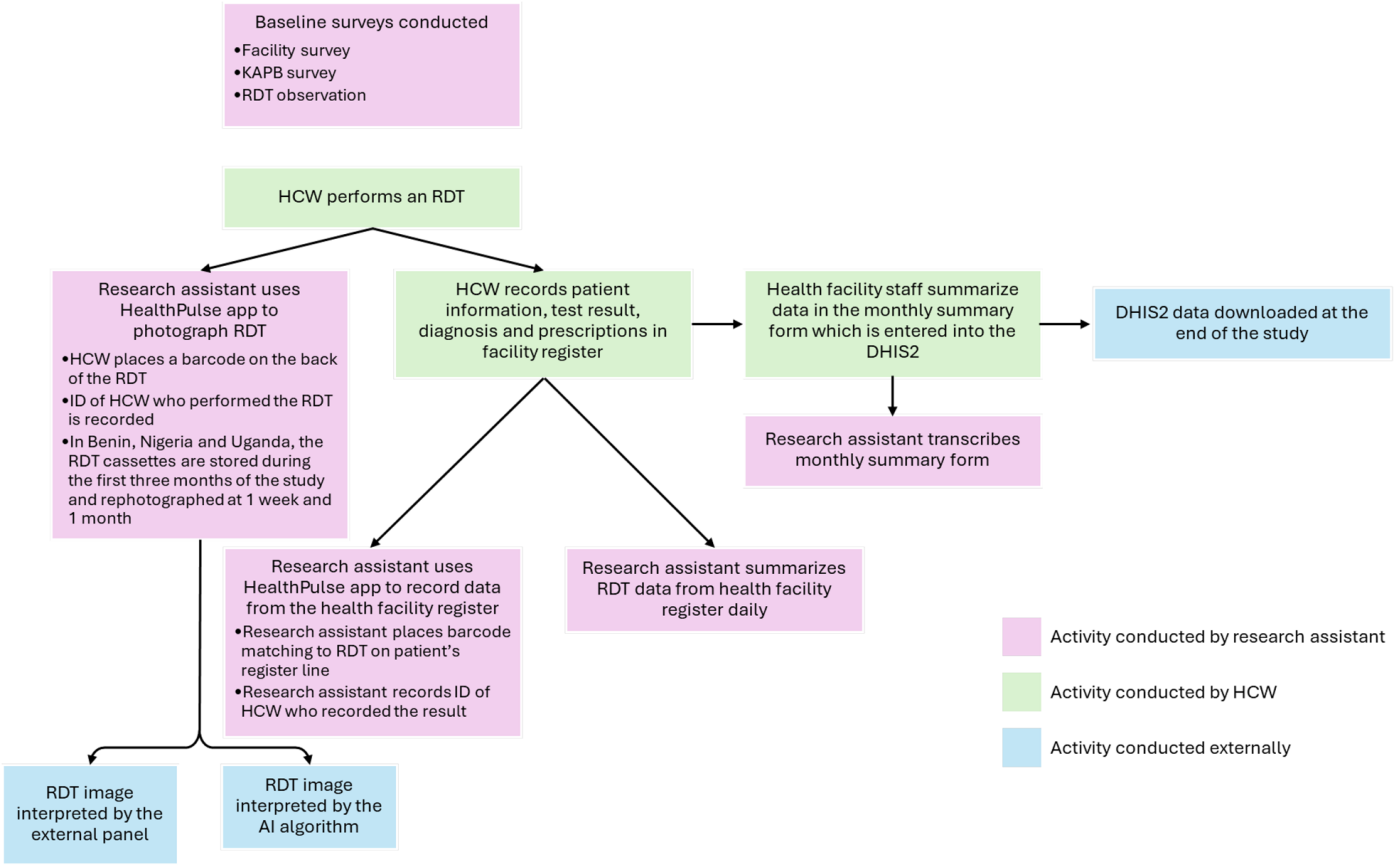
Schematic describing key data collection activities and information flows in each country participating in the MaCRA study. *AI* artificial intelligence, *DHIS2* District Health Information System 2, *HCW* healthcare worker, *ID* identification, *KAPB* Knowledge, attitudes, perceptions and behaviours, *RDT* rapid diagnostic test

The main data collection activities and information flows that are described below are summarized in Fig. 2.

#### Baseline surveys

##### Facility survey

A survey was conducted at baseline to record the operating characteristics of each study facility through an interview with the facility officer in-charge and direct observation. The survey included: a count of the HCWs working in the facility by position and qualifications; presence of registers, guidelines and job aids; availability of laboratory diagnostic tests; first- and second-line antimalarial medicines; stockouts of medicines and supplies over the previous 3 months (a stockout was described as at least seven consecutive days without the medicine or supply); and status of infrastructure such as electricity and internet connection.

##### Knowledge, attitudes, perceptions and behaviour survey and RDT performance evaluation

At baseline, all HCWs in the study health facilities who had current or potential responsibility for testing, interpretation, or recording of RDT results or treatment of patients with malaria were administered a knowledge, attitudes, perceptions and behaviour (KAPB) survey. The survey gathered data on: HCWs training and experience; knowledge of malaria transmission and case management; attitudes towards RDTs; perceptions of the accuracy of RDTs; behaviours associated with prescription of antimalarial medicines; and subjective norms with respect to RDTs, malaria case management and surveillance practices.

HCWs who routinely performed RDTs at the facility were observed conducting an RDT in the presence of a research assistant. Their performance was assessed using a standardized 19-point checklist derived from a validated tool, covering biosafety, procedural steps, and result interpretation [18]. HCWs who joined study facilities after the start of the study were also observed performing an RDT and completed the KAPB survey, following written informed consent.

Each HCW who participated in the KAPB survey was assigned a unique, random identification code that was recorded with their name in a notebook kept locked outside of working hours. This unique identification code was used in the HealthPulse application to link RDTs to the HCWs who performed or recorded results and their responses to the KAPB survey and RDT performance evaluation. To preserve the anonymity of results, all notebooks were gathered and destroyed at the end of the study after data cleaning was completed.

#### Collection of RDT images, results and patient data

Research assistants captured photographs of all RDTs performed at the facility as soon as possible after interpretation by HCWs, without disrupting patient care. After placing a label with a unique preprinted barcode on the back of each RDT, they opened the HealthPulse app, scanned the barcode and followed instructions to take a well-lit photo of the RDT using the smartphone camera. If the app’s IQA signaled a problem with the image, the research assistant was instructed to retake the photo, although the original photo was retained if a new image was not taken. The research assistant entered the unique identification code for the HCW who performed the RDT. Images were automatically uploaded to a server in South Africa before being sent to the external panel for interpretation.

A matching barcode was affixed next to the entry in the health facility register corresponding to the patient for whom the RDT was performed. Research assistants used the HealthPulse app to scan the barcode and then entered the unique identification code of the HCW who recorded the RDT result in the register. Research assistants also recorded the patient’s age, sex, RDT result, clinical diagnosis, and prescription of antimalarials and antibiotics.

#### Collection of RDT images over time

For the first 3 months of the study in Benin, Nigeria and Uganda, research assistants stored RDT cassettes in paper envelopes and cardboard boxes at ambient temperature and humidity in health facilities after their interpretation by the HCW. RDT cassettes were rephotographed 1 week and 1 month after the initial interpretation. At each time point, the barcode affixed to the back of the cassette was scanned to enable consistent matching of images over time. Images were sent to the external panel for interpretation. Following the final image capture, RDT cassettes were disposed of in accordance with national guidelines.

#### Objective interpretation of RDT results

RDT images were sent to a trained and quality-controlled external panel of 20 RDT interpreters who independently reviewed randomly assigned images and were blinded to any metadata or patient data. Panelists identified the RDT product in the image and interpreted the RDT result as positive, negative, or invalid by determining the presence or absence of the control and test lines. A result was classified as indeterminate if the panelists were unable to visualize the control or test lines due to an artifact obstructing the image or due to deficiencies in image quality. Panelists flagged problematic photo conditions such as blur, glare, darkness, skew, and presence of multiple RDTs in a single photo, identified excess blood in the result window and noted when test lines were faint. Panelists were trained on both good and poor-quality photos and were required to pass a test that involved correctly interpreting and labeling a set of 50 images. Quality control of RDT interpretations involved two stages: an initial review of 100% of images and a second stage that reviewed a random selection of 30% of images that had passed the first stage. If a reviewer identified errors in interpretation or labeling anomalies at any stage, the images were sent back to the pool for a fresh interpretation.

For the first 2–4 weeks of the study in each country, all images were reviewed by three panelists. After validation of the results indicated that there was near perfect agreement among panelists (Fleiss’ kappa > 0.99 for each country, Supplementary Table 2), each RDT image was reviewed by a single panelist, randomly assigned. Panel interpretations of the RDT results were not made available to the study investigators (apart from SC) until the end of the study except for the limited initial validation.

#### Health facility registers, monthly summary forms and DHIS2 data

Research assistants summarized data from health facility registers each day, including the number of malaria RDTs performed and their results, and entered the information into a KoboToolbox data form. Each month, research assistants digitized the aggregated malaria results included on the MSF that was prepared for entry into the DHIS2 by health facility staff. After the study was completed, DHIS2 data on the number of positive RDT results and the total number of RDTs administered were extracted for each study and control facility from January 2021 to December 2023. This period includes 30 months prior to study initiation and the 4- to 6-month study period.

### Sample size

The sample size for the main objective was based on the level of precision for estimating Cohen’s kappa (κ) for an individual HCW [19]. A range of κ scores between 0.7 and 0.9 was assumed and the number of RDTs required per HCW was calculated for different levels of precision. The probability of a positive rating was estimated by the TPR. Assuming TPRs between 30, 70%, the maximum sample size needed to calculate 95% confidence intervals for κ with a width of no more than 0.2 was 236 RDTs per HCW. It was estimated that individual HCWs would likely interpret between 40 and 80 RDTs each month, for a possible range of 320–480 observations per HCW over the course of the evaluation, which would be sufficient for a precise measurement of κ at both the HCW and health facility level. The number of study facilities was fixed at 16 due to budget limitations.

### Data management

The geographic coordinates of each study facility were recorded and used to calculate average *P. falciparum* parasite prevalence for children 2–10 years (PfPR_2–10_) within 5 km of the facility using the malariaAtlas package in R (R Foundation for Statistical Computing, Vienna, Austria) [20].

All RDTs whose results were classified as indeterminate by the external panel were discarded as these resulted from errors in the image. When a HCW recorded a positive RDT result in the register but the external panel interpreted the result as either negative or invalid, the HCW result was classified as ‘misrecorded as positive.’ When a HCW recorded a negative RDT result but the external panel interpreted the result as either positive or invalid, the HCW result was classified as ‘misrecorded as negative.’ There were no instances of a HCW recording an invalid RDT result.

HCWs who performed RDTs were classified as clinical (e.g. nurse, medical doctor, midwife, community health worker) or non-clinical staff (e.g. cleaner, guard, volunteer). Questions of perceptions or beliefs based on the Likert scale were dichotomized into agree (agree or strongly agree) or do not agree (neutral, disagree or strongly disagree). The 19-point RDT performance score was divided into terciles based on the distribution of scores in each country.

### Statistical analysis

Statistical methods for the primary and secondary objectives are described briefly while more detailed statistical methods will be included in individual reports. Statistical analyses were conducted in R.

#### Accuracy of recorded RDT results

Cohen’s kappa coefficient (κ) was used to measure agreement between RDT results recorded by HCWs in facility registers and those interpreted by the external panel. To produce an overall summary measure of agreement across health facilities, a random-effects meta-analysis was conducted in R using a bespoke function based on the kappa2 function from the irr package in R. Facility-level kappa estimates were weighted by the inverse of their variance and 95% confidence intervals (CI) were calculated [21, 22].

#### Factors associated with results misrecorded as positive or negative

Multinomial logistic regression was used to model the odds ratio (OR) of an RDT result being misrecorded as positive or negative based on characteristics of the facility, HCW and RDT, adjusted for health facility. 95% CI were calculated for all OR with robust standard errors adjusted for clustering of RDT results by HCW.

#### Stability of RDT results

Simple descriptive statistics were used to calculate the proportion of initially positive or negative RDTs that retained their original result 1 month (i.e. 25–35 days) after the initial interpretation. Positive and negative predictive values (PPV and NPV, respectively) of the 1-month outcome for the original result were calculated and 95% CI were calculated using the Wilson score method for binomial proportions using the binom package in R [23].

#### Comparing numbers of positive RDTs by data source

The weighted absolute percentage error (WAPE) was used to quantify the discrepancy between RDT data reported from the DHIS2 and those recorded in the health facility registers. WAPE was calculated as the sum of the absolute differences between the two data sources divided by the total from the health facility registers, expressed as a percentage. The formula is given as [24]:

$$WAPE = \left( {\frac{{\sum\nolimits_{{m = 1}}^{n} {\left| {y_{{m - }} \hat{y}_{m} } \right|} }}{{\sum\nolimits_{{m = 1}}^{n} {y_{m} } }}} \right){\mkern 1mu} \times {\mkern 1mu} 100$$

WAPE was calculated for each facility as the sum of the absolute values of the differences between the health facility register and the DHIS2 each month divided by the total number of observations in the register over the time period. To facilitate interpretation, a WAPE-based metric of reporting accuracy termed the weighted aggregate data reporting accuracy (WADRA) was calculated as 100%—WAPE. The accuracy of each facility was categorized as high (WADRA ≥ 85%), moderate (70, 84%) or low (< 70%) based on previously published thresholds [24]. The analysis included only months for which the study team was in place for the entire period.

#### Measuring the observer effect on test positivity rates

A difference-in-difference analysis was conducted comparing the TPR measured by RDTs reported by the DHIS2 before and after the study was initiated between study and control health facilities. A negative binomial, mixed effects linear regression model was constructed using integrated nested Laplace approximation with health facility-month as the unit of analysis, the number of positive RDT results as the dependent variable and log-transformed counts of RDTs as the model offset [25]. Risk ratios (RR) with 95% credibility intervals (CI) were calculated.

### Ethical issues

Written, informed consent was obtained from HCWs who participated in the KAPB survey and were observed administering an RDT. HCWs were informed that anonymous and unique identifiers would be used in the HealthPulse app to link information from the KAPB survey and RDT observation to RDTs performed or recorded by HCWs. No patients were consented by the study team as the RDT images and data from the health facility registers were recorded anonymously and obtained from secondary data sources, respectively. No personally identifiable information was obtained from patient records. Patients were managed by HCWs in accordance with national case management guidelines.

The PATH institutional review board approved the multi-country study protocol. In Benin, the Comité National d’Ethique pour la Recherche en Santé provided approval. In Côte d’Ivoire, the Comité National d'Éthique des Sciences de la Vie et de la Santé reviewed and approved the study. In Nigeria, approval was received from Oyo State Ministry of Health Research Ethics Committee, Sokoto State Health Research Ethics Committee and the National Health Research Ethics Committee of Nigeria. The Uganda National Council for Science and Technology and Vector Control Division-Research & Ethics Committee both reviewed and approved the study in Uganda.

## Results

The key results of the MaCRA study are presented in this report while more detailed results will be presented in separate publications.

### Accuracy of recorded RDT results

Between June and December 2023, 110,843 RDTs were observed across the four countries; after matching images with patient records, 102,327 (92.3%) RDTs were available for analysis. TPRs calculated from RDT results recorded by HCWs in health facility registers were lowest in Nigeria (48.0%) and highest in Côte d’Ivoire (63.8%) and were higher than those calculated from the external panel by between 2.7 and 5.5 percentage-points (pct-pts; Table 1). There were no invalid results reported in the health facility registers while the external panel interpreted 227 RDTs (0.2%) as invalid. The level of interrater agreement as measured by κ was high for each country and ranged from 0.80 (95% CI 0.75, 0.85) in Nigeria to 0.88 (95% CI 0.84, 0.92) in Benin. Among results not in agreement between HCWs and the external panel, HCWs were more likely to misrecord RDT results as positive than negative; the proportion of results misrecorded as positive ranged from 5.1% in Benin to 7.3% in Uganda while results misrecorded as negative ranged from 0.7% in Benin to 3.7% in Nigeria (Table 1).

**Table 1:** Number of rapid diagnostic tests observed in the study and measurement of interrater agreement between results recorded in facility registers and an external panel by country, 2023

Country	No. RDTs observed and matched with patient data	Positive RDTs recorded by HCWs n (TPR %)	Positive RDTs recorded by the external panel n (TPR %)	Cohen’s kappa (95% CI)	Results misrecorded as positive n (%)	Results misrecorded as negative n (%)
Benin	35,720	19,480 (54.5)	17,913 (50.1)	0.88 (0.84, 0.92)	1805 (5.1)	244 (0.7)
Côte d’Ivoire	11,161	7126 (63.8)	6662 (59.7)	0.82 (0.76, 0.87)	662 (5.9)	218 (2.0)
Nigeria	18,319	8792 (48.0)	8302 (45.3)	0.80 (0.75, 0.85)	1129 (6.2)	675 (3.7)
Uganda	37,137	22,952 (61.8)	20,913 (56.3)	0.81 (0.78, 0.84)	2698 (7.3)	696 (1.9)

*CI* confidence interval, *HCWs* healthcare workers, *N* number, *RDTs* rapid diagnostic tests, *TPR* test positivity rate

### Factors associated with misrecording RDT results

There were no factors consistently and statistically associated with misrecording results as positive across all countries (Table 2). However, patients aged 15 years and older were consistently more likely to have results misrecorded as positive than patients 0–4 years old in Benin, Côte d’Ivoire and Uganda although not in Nigeria. In Benin and Côte d’Ivoire but not the other two countries, children 5–14 years old were also more likely than children 0–4 years old to have their RDT result misrecorded as positive.

**Table 2:** Factors associated with rapid diagnostic test results misrecorded as positive or negative in a multinomial logistic regression model adjusted for health facility, 2023

Variable	Benin n = 35,720 OR (95% CI)	Cote d’ Ivoire n = 11,161 OR (95% CI)	Nigeria n = 18,319 OR (95% CI)	Uganda n = 37,137 OR (95% CI)
Misrecorded as positive				
Patient sex				
Female	1.12 (0.97, 1.28)	1.09 (0.91, 1.31)	**1.13 (1.02, 1.24)**	1.01 (0.93, 1.11)
Male	1.0	1.0	1.0	1.0
Patient age (years)				
0–4	1.0	1.0	1.0	1.0
5–14	**1.47 (1.24, 1.75)**	**1.39 (1.11, 1.75)**	1.00 (0.82, 1.22)	1.02 (0.90, 1.15)
15 +	**2.71 (2.34, 3.15**)	**1.92 (1.64, 2.25)**	1.11 (0.90, 1.36)	**1.40 (1.22, 1.61)**
HCW sex				
Female	1.17 (0.91, 1.51)	1.30 (0.84, 2.02)	0.70 (0.45, 1.10)	0.98 (0.87, 1.12)
Male	1.0	1.0	1.0	1.0
HCW age (years)				
< 30	1.0	1.0	1.0	1.0
30–39	**0.76 (0.59, 0.98)**	**2.15 (1.22, 3.78)**	0.90 (0.58, 1.40)	**0.76 (0.60, 0.97)**
40 +	**0.48 (0.36, 0.63)**	**2.11 (1.23, 3.61)**	0.87 (0.64, 1.20)	**0.78 (0.62, 0.98)**
HCW occupation category				
Clinical staff	0.87 (0.71, 1.07)	1.10 (0.65, 1.85)	0.77 (0.56, 1.05)	1.02 (0.76, 1.38)
Non-clinical staff	1.0	1.0	1.0	1.0
HCW highest qualification achieved				
Secondary school or below	1.0	1.0	1.0	1.0
University	0.84 (0.66, 1.08)	1.45 (0.88, 2.40)	1.19 (0.91, 1.56)	0.89 (0.71, 1.13)
HCW years of experience				
0–4	1.0	1.0	1.0	1.0
5 +	**0.78 (0.64, 0.96)**	1.17 (0.77, 1.78)	0.89 (0.71, 1.13)	**0.72 (0.58, 0.89)**
HCW frequency of administering RDT				
Very often	0.55 (0.28, 1.09)	0.93 (0.66, 1.32)	1.02 (0.69, 1.53)	**0.47 (0.29, 0.77)**
Once in a while to often	0.46 (0.21, 1.02)	1.50 (0.69, 3.25)	**0.26 (0.11, 0.61)**	**0.49 (0.30, 0.81)**
Never	1.0	1.0	1.0	1.0
HCW considers RDTs easy to perform				
Agree	1.09 (0.77, 1.52)	0.80 (0.42, 1.53)	**0.52 (0.32, 0.84)**	1.18 (0.79, 1.76)
Do not agree	1.0	1.0	1.0	1.0
HCW has sufficient time to perform RDTs				
Agree	1.39 (1.00, 1.95)	1.25 (0.57, 2.74)	0.54 (0.39, 0.75)	1.13 (0.89, 1.43)
Do not agree	1.0	1.0	1.0	1.0
HCW could provide antimalarial to the patient even if RDT negative				
Yes	1.02 (0.65, 1.60)	**2.01 (1.01, 3.98)**	0.69 (0.46, 1.05)	1.03 (0.92, 1.15)
No	1.0	1.0	1.0	1.0
HCW received training in RDTs				
Yes	1.21 (0.87, 1.70)	0.64 (0.29, 1.38)	0.94 (0.67, 1.33)	1.02 (0.85, 1.22)
No	1.0	1.0	1.0	1.0
Supervisor observed HCW perform an RDT during past visit				
Yes	1.22 (0.87, 1.73)	**0.41 (0.27, 0.62)**	2.81 (0.81, 9.71)	1.06 (0.87, 1.29)
No	1.0	**1.0**	1.0	1.0
RDT proficiency tercile				
Low	1.0	1.0	1.0	1.0
Middle	1.32 (0.86, 2.02)	**3.99 (1.16, 13.68)**	1.34 (0.98, 1.83)	0.99 (0.70, 1.39)
High	**1.56 (1.06, 2.31)**	**3.64 (1.02, 12.97)**	0.87 (0.65, 1.17)	0.87 (0.63, 1.21)
Misrecorded as negative				
Patient sex				
Female	0.94 (0.70, 1.25)	0.97 (0.77, 1.23)	1.00 (0.82, 1.21)	1.07 (0.91, 1.25)
Male	1.0	1.0	1.0	1.0
Patient age (years)				
0–4	1.0	1.0	1.0	1.0
5–14	0.74 (0.51, 1.08)	1.04 (0.75, 1.44)	0.93 (0.73, 1.18)	1.07 (0.85, 1.35)
15 +	**1.83 (1.31, 2.55)**	**1.87 (1.49, 2.34)**	1.17 (0.96, 1.41)	**1.74 (1.41, 2.16)**
HCW sex				
Female	1.11 (0.68, 1.81)	0.92 (0.67, 1.25)	1.11 (0.78, 1.58)	1.00 (0.85, 1.17)
Male	1.0	1.0	1.0	1.0
HCW age (years)				
< 30	1.0	1.0	1.0	1.0
30–39	0.86 (0.49, 1.53)	0.82 (0.50, 1.33)	1.01 (0.81, 1.27)	1.07 (0.87, 1.32)
40 +	1.16 (0.60, 2.22)	0.75 (0.47, 1.20)	1.32 (0.94, 1.86)	1.05 (0.85, 1.30)
HCW occupation category				
Clinical staff	1.09 (0.74, 1.59)	1.25 (0.77, 2.03)	0.88 (0.61, 1.29)	1.29 (0.94, 1.77)
Non-clinical staff	1.0	1.0	1.0	1.0
HCW highest qualification achieved				
Secondary school or below	1.0	1.0	1.0	1.0
University	0.73 (0.31, 1.75)	0.91 (0.52, 1.61)	0.94 (0.62, 1.41)	1.27 (0.96, 1.67)
HCW years of experience				
0–4	1.0	1.0	1.0	1.0
5 +	1.01 (0.46, 2.21)	0.76 (0.57, 1.02)	1.26 (1.00, 1.60)	1.19 (0.95, 1.50)
HCW frequency of administering RDT				
Very often	0.53 (0.19, 1.46)	0.99 (0.55, 1.80)	1.57 (0.95, 2.57)	0.49 (0.09, 2.60)
Once in a while to often	**0.09 (0.01, 0.67)**	0.56 (0.23, 1.40)	1.56 (0.76, 3.20)	0.36 (0.07, 1.94)
Never	1.0	1.0	1.0	1.0
HCW considers RDTs easy to perform				
Agree	1.25 (0.71, 2.19)	0.58 (0.26, 1.30)	1.02 (0.63, 1.68)	**0.56 (0.45, 0.69)**
Do not agree	1.0	1.0	1.0	1.0
HCW sufficient time to perform RDTs				
Agree	1.10 (0.71, 1.71)	**0.66 (0.45, 0.96)**	0.68 (0.36, 1.29)	1.02 (0.70, 1.50)
Do not agree	1.0	1.0	1.0	1.0
HCW could provide antimalarial to patient with negative RDT				
Yes	1.56 (0.85, 2.86)	0.98 (0.59, 1.63)	0.97 (0.64, 1.45)	0.94 (0.76, 1.17)
No	1.0	1.0	1.0	1.0
Received training in RDTs				
Yes	1.05 (0.71, 1.55)	0.68 (0.30, 1.53)	0.80 (0.62, 1.03)	1.00 (0.83, 1.20)
No	1.0	1.0	1.0	1.0
Supervisor observed HCW perform an RDT during past visit				
Yes	0.96 (0.68, 1.36)	1.13 (0.85, 1.50)	1.77 (0.72, 4.36)	**0.73 (0.60, 0.89)**
No	1.0	1.0	1.0	1.0
RDT proficiency tercile				
Low		1.0	1.0	1.0
Middle	1.0	0.53 (0.23, 1.24)	1.05 (0.72, 1.53)	1.33 (1.01, 1.75)
High	1.58 (0.69, 3.66)	0.41 (0.17, 0.97)	0.87 (0.56, 1.33)	0.96 (0.69, 1.33)

*CI* confidence interval, *HCW* healthcare worker, *OR* odds ratio, *RDTs* rapid diagnostic tests

Bolded results indicate 95% confidence intervals that do not include the null value

In Benin and Uganda, older HCWs and those with more years of experience were less likely to misrecord RDT results as positive (Table 2). In Côte d’Ivoire, however, older HCWs were more likely to misrecord RDT results as positive. Additionally in Côte d’Ivoire, HCWs who answered “Yes” to the statement, “Might you provide an antimalarial medicine to a patient even if their RDT returns a negative result?” and those who were in the top two terciles of RDT proficiency were more likely to misrecord RDT results as positive. HCWs in Côte d’Ivoire whose supervisor had observed them performing an RDT were less likely to misrecord results as positive. In Nigeria, female patients were more likely to have their RDT results misrecorded as positive than male patients, while HCWs that considered RDTs easy to perform were less likely to misrecord results as positive. In Uganda, older HCWs, those with more years of experience and those who frequently administered RDTs were less likely to misrecord a result as positive. HCWs with RDT proficiency scores in the top two terciles were conversely more likely to misrecord an RDT as positive in Benin as those in the lowest, but there was no statistical association between RDT proficiency scores and misrecording results as positive in any other country.

Few factors were statistically associated with misrecording results as negative (Table 2). Patients 15 years and older were more likely than patients aged 0–4 years to have results misrecorded as negative in Benin (OR 1.83, 95% CI 1.31, 2.55), Côte d’Ivoire (OR 1.87, 95% CI 1.49, 2.34) and Uganda (OR 1.74, 95% CI 1.41, 2.16) but not Nigeria. In Côte d’Ivoire, HCWs who felt they had sufficient time to perform RDTs were less likely to misrecord results as negative, while in Uganda, HCWs who considered RDTs easy to perform and those whose supervisor observed their performance of an RDT during a past visit were less likely to misrecord results as negative.

### Stability of RDT cassette results over time

There were 45,445 stored RDT cassettes that were re-imaged 1 month after they were administered. At the time of administration, there were 25,484 (56.1%) positive, 19,899 (43.8%) negative, and 62 (0.1%) invalid results. At the end of 1 month, there were 25,241 (55.5%) positive, 20,192 (44.4%) negative and 12 (0.0%) invalid results. Over the 1-month period, 1245 (4.9%) of the initially positive results changed to negative, 959 (4.8%) of the initially negative results changed to positive, 43 (69.4%) of the invalid results converted to positive, and 7 (11.3%) of the invalid results converted to negative. The PPV of a positive outcome at 1 month was 96.0% (95% CI 95.9, 96.4), the NPV was 93.8% (95% CI 93.4, 94.1) and the equivalent metric for an invalid test was 100% (95% CI 67.5, 100).

### Comparing numbers and results of RDTs between the DHIS2 and health facility registers

**Fig. 3:**
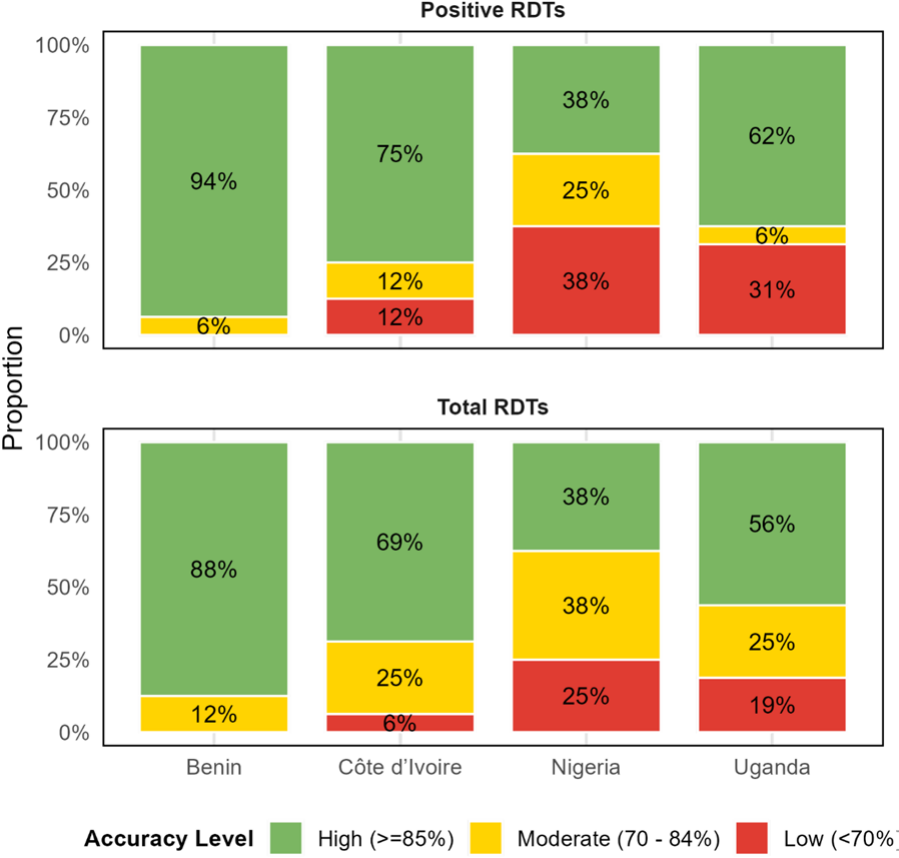
Proportion of health facilities with high, moderate or low levels of WAPE-based aggregate data reporting accuracy by indicator and country, 2023

In Benin, nearly all (94%) health facilities were categorized as having a high level of reporting accuracy for their DHIS2 data with 6% categorized with a moderate level of accuracy (Fig. 3). In Nigeria and Uganda, between 19 and 38% of facilities were classified as having low reporting accuracy, depending on the outcome assessed. Côte d’Ivoire had few facilities with low reporting accuracy and between 12 and 25% of facilities with moderate accuracy.

### Measuring the observer effect on test positivity rates

Comparing the periods before and after the start of the study, TPRs reported by the DHIS2 declined more in study facilities than in control facilities across all countries (Table 3). However, the difference was only statistically significant in Côte d’Ivoire. In Côte d’Ivoire, TPRs in study facilities decreased by 20.4 pct-pts more than in control facilities and modeled results estimated an RR of 0.80 (95% credibility interval 0.76, 0.84). While Nigeria and Uganda also showed larger reductions in TPRs in study facilities compared to control facilities (10.2 pct-pts and 6.4 pct-pts, respectively), the credibility intervals for these estimates included the null, indicating that the differences were not statistically significant. Benin exhibited the smallest difference-in-difference, with a 1.3 pct-pt greater decline in TPRs in study facilities compared to control facilities, but this decrease was not statistically significant.

**Table 3:** Changes in observed test positivity rates before and after the MaCRA study was initiated in study and control facilities and modeled difference-in-difference results, January 2021, December 2023

Country	TPR before study start		TPR after study start		Difference of differences	Modeled results
	Study facilities	Control facilities	Study facilities	Control facilities	Pct-pts	RR (95% CI)
Benin	70.1	69.8	49.1	50.1	1.3	0.98 (0.87, 1.11)
Côte d’Ivoire	85.3	86.1	68.2	89.3	20.4	0.80 (0.76, 0.84)
Nigeria	72.7	69.0	58.5	64.8	10.2	0.90 (0.79, 1.04)
Uganda	67.9	66.2	58.4	63.1	6.4	0.92 (0.83, 1.00)

*CI* credibility interval, *Pct-pts* percentage points, *RR* risk ratio, *TPR* test positivity rate

## Discussion

We compared RDT results recorded by HCWs in health facility registers with those independently interpreted by an external panel reviewing images of the same RDTs and found strong levels of agreement as measured using Cohen’s kappa. However, agreement was not perfect and there remained an important proportion of RDT results that were inaccurately recorded in health facility registers. Between 5.1 and 7.3% of RDT results were originally negative or invalid but misrecorded in health facility registers as positive, while between 0.7 and 3.7% of results that were originally positive or invalid were misrecorded as negative. Overall, the proportion of RDT results that were recorded inaccurately in health facility registers ranged from 5.8% in Benin to 9.9% in Nigeria. Although the proportion of RDTs interpreted as invalid by the external panel was very low (0.2%), all were nonetheless treated as valid by healthcare workers and used to guide clinical decision-making. Taken together, these findings suggest that RDT results misrecorded as positive were consistently more frequent than those misrecorded as negative, suggesting that misrecording RDT results as positive was not a random error but may reflect a systematic influence on RDT recording behaviour. Given the emphasis placed by donors and ministries of health to justify the quantity of antimalarial treatments dispensed with the number of confirmed malaria cases, it is plausible that HCWs may misrecord negative results as positive to align with treatment decisions that do not adhere to RDT results.

Factors underlying HCWs’ non-adherence to RDT results have been extensively examined in previous studies, and include a lack of trust in RDTs, patient demand for antimalarial medication and the lack of alternative diagnostics or treatments [8, 9, 26]. Patient age was the only factor consistently associated with the misrecording of results as positive in three of the four countries. Although the consequences of a missed malaria infection may be more severe in young children, the odds of negative or invalid RDT results being misrecorded as positive were higher among patients aged 15 years and older than among younger children. Several qualitative studies have documented the pressure patients may place on HCWs to prescribe antimalarials despite negative RDT results, and this pressure may be more pronounced among older patients [9, 27, 28]. Other factors previously identified in the literature to be associated with non-adherence to RDT results such as professional cadre, level of training and perceptions of RDTs were not associated with the misrecording of negative results as positive in this study [26, 29–31].

Results misrecorded as negative were less common and may arise from a failure to wait long enough for an RDT to fully develop before interpreting the result. Additionally, vision limitations may make the lines on RDTs difficult to see. HCWs may also misrecord a positive result as negative if they are concerned about a prior malaria infection and want to avoid potential overtreatment [6]. However, not providing antimalarials to those who are symptomatic and test positive for malaria by RDT risks development of severe disease or death and HCWs should be counseled on appropriate practices.

In addition to errors resulting from the misrecording of individual RDT results, inaccuracies in RDT data may occur during the processes of data aggregation, summarization, and manual entry into DHIS2. The accuracy of total and positive RDT numbers reported to the DHIS2 was high in Benin, lower in Côte d’Ivoire, and very low in a substantial proportion of study facilities in Nigeria and Uganda. Implementing a review process to verify MSFs and ensure the accuracy of data aggregation at the health facility level may help address data concordance issues in Nigeria and Uganda.

In Côte d’Ivoire, the start of this study was associated with a substantial decline in TPR in study facilities, whereas matched control facilities experienced a small increase. In Nigeria and Uganda, study facilities also exhibited decreases in TPR compared to controls but declines were not statistically significant. In contrast, Benin experienced only a minimal, non-significant decline in TPR. The marked decrease in Côte d’Ivoire suggests a potential change in HCW behaviour triggered by the start of the study. It is possible that increased attention to RDT results during the study may have prompted HCWs to record RDT outcomes more accurately in facility registers, particularly by reducing the frequency of results misrecorded as positive. A similar behavioural shift may have occurred earlier in Benin. In January 2023, 6 months before the study began, Benin launched a national program for monthly RDT validation using stored RDT cassettes [32]. The decline in TPR following that program suggests that HCW practices around recording RDT results may have already improved by the time the study commenced.

While manufacturers recommend that RDTs be interpreted within 15–25 min of sample application, the findings from this study indicate that most RDT results remain stable for up to 1 month under routine storage conditions. This suggests that stored RDT cassettes may serve as reliable source documents for validating health facility register data. Several countries have begun to adopt this practice; in addition to Benin, Nigeria has implemented RDT archiving and validation in certain states [33]. The findings from this study generally support the use of stored RDTs for validation purposes. However, caution is warranted, as a small proportion of RDT results may not remain stable over time, and different RDT products from those assessed in this study may not have the same level of stability.

This study had several limitations. First, the reference standard used to assess the accuracy of recorded RDT results was based on photographs of the RDTs. As cameras may not capture all visual details observable to the human eye, this reference standard was imperfect [34]. However, given the large number of RDTs assessed, this limitation is not likely to have meaningfully affected the overall findings. Second, HCW characteristics were linked to the individual who recorded the RDT result in the facility register as that person was considered to have made the final decision on the RDT result to be recorded. In some cases, however, the decision as to which RDT result to record may have been made by a different HCW, introducing potential non-differential misclassification and attenuating associations between HCW characteristics and misrecording. Third, differences in site selection criteria and study implementation across countries may have influenced country-specific results in ways that could not be fully accounted for. Fourth, the strong level of agreement on RDT results found in Côte d’Ivoire, and to a lesser extent in Nigeria and Uganda, may be an overestimate due an observer bias. Finally, the study was limited in duration, number of facilities, and geographic scope, and thus may not be fully representative of the broader health system context in each country.

## Conclusions

Although RDT results recorded in health facility registers demonstrated a strong level of agreement with external, objective panel reviews of RDT images in Benin, Côte d’Ivoire, Nigeria, and Uganda, inaccuracies in recording results remain a concern. The observed change in TPR following the start of the study in Côte d’Ivoire, likely due to an observer effect generated by increased attention to RDTs, suggests that some HCWs can be motivated to improve the accuracy of malaria data. The experience in Benin, where monthly data validation meetings using stored RDT cassettes have been held at health zone level nation-wide since January 2023, highlights a promising approach to enhancing the accuracy of RDT results, and results from this study document the stability of results on RDT cassettes over at least a 1 month period. Prospective testing of monthly data validation using stored RDT cassettes and evaluation of its acceptability to HCWs, feasibility of implementation, impact and duration of effect could provide national malaria programs with an evidence-based strategy to improve and sustain accuracy of routine malaria surveillance data.

## Supplementary Information

Supplementary material 1: Table 1. Description of RDT products recognized by the HealthPulse application. Table 2. Fleiss’ kappa score calculated to measure interrater agreement on RDT results among three external panelists by country, 2023

## Data Availability

The datasets used and/or analyzed during the current evaluation can be provided by the corresponding author on reasonable request.

## Abbreviations

AI: Artificial intelligence
CI: Confidence interval or credibility interval
DHIS2: District health information system 2
HCW: Healthcare worker
HMIS: Health management information system
HRP2: Histidine-rich protein 2
IQA: Image quality assurance
IRB: Institutional review board
KAPB: Knowledge, attitudes, perceptions and behaviours
MOH: Ministry of health
N: Number
OPD: Outpatient department
OR: Odds ratio
PCR: Polymerase chain reaction
Pct-pts: Percentage-points
pLDH: Parasite lactate dehydrogenase
PMI: President’s Malaria Initiative
RDT: Rapid diagnostic test
RR: Risk ratio
SE: Standard error
SSA: Sub-Saharan Africa
TPR: Test positivity rate
WAPE: Weighted average percentage error
WHO: World Health Organization
