# Supplementary material for: Assessing the accuracy of the recording and reporting of malaria rapid diagnostic test results in four African countries: methods and key results
Source: Malar J. 2025 Jul 1;24:206. doi: 10.1186/s12936-025-05459-7 (PMC12219610; doi:10.1186/s12936-025-05459-7)
Supplement: Supplementary file 1 — Supplementary material 1: Table 1. Description of RDT products recognized by the HealthPulse application. Table 2. Fleiss’ kappa score calculated to measure interrater agreement on RDT results among three external panelists by country, 2023 [file 12936_2025_5459_MOESM1_ESM.docx]

Supplementary table 1. Description of RDT products recognized by the HealthPulse application.

| **RDT product (manufacturer)** | **Referred to as:** | **RDT cassette format** |
| --- | --- | --- |
| RDT cassettes with a single test line to detect *Plasmodium falciparum*-specific histidine-rich protein 2 (HRP2) | | |
| AdvDx Malaria Pf Rapid Malaria Ag Detection Test (Advy Chemical, Mumbai, India) [35] | AdvDx Malaria Pf | 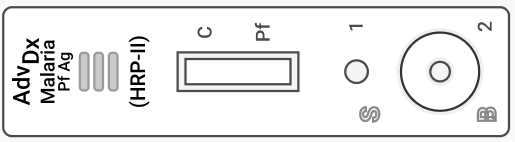 |
| Bioline MALARIA Ag P.f  (Abbott, IL USA) [36]  *Note: The same cassette previously was manufactured as SD Bioline.* | Bioline Malaria Pf | 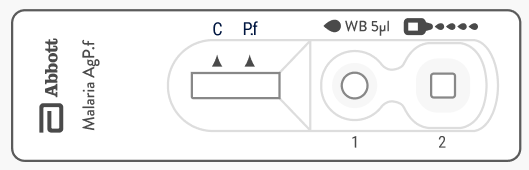  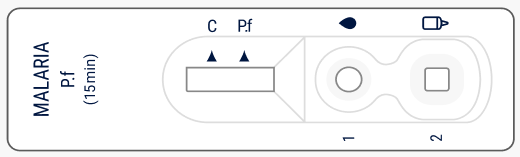 |
| CareStart Malaria Pf (HRP2) Ag RDT  (Access Bio Inc, NJ USA)  *Note: This product has been de-listed by WHO prequalification.* | CareStart Malaria Pf | 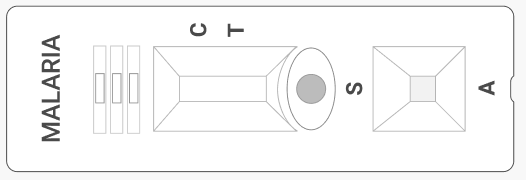 |
| First Response Malaria Antigen *P. falciparum* (HRP2) Card Test (Premier Medical Corporation Ltd, Gujarat, India) [37] | First Response Malaria Pf | 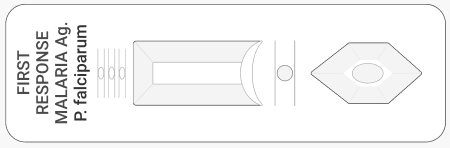 |
| ParaHIT f Ver. 1.0 Rapid Test for *P. falciparum* Malaria Device  (Arkray Healthcare Prvt Ltd, Mumbai, India) [38] | ParaHIT Malaria Pf | 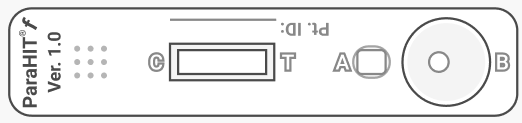 |
| STANDARD Q Malaria P.f Ag Test (SD Biosensor, Gyeonggi-do, Republic of Korea) [39] | Standard Q Malaria Pf | 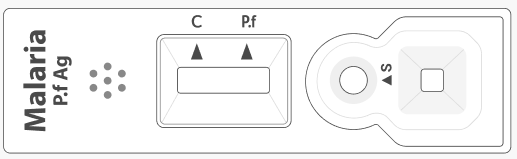 |
| RDT cassettes with two test lines to detect *P. falciparum* HRP2 and *P. falciparum*-specific lactate dehydrogenase (pLDH) | | |
| Bioline Malaria Ag P.f (HRP2/pLDH) (Abbott, IL USA) [40] | Bioline Malaria Pf (HRP2/pLDH) | 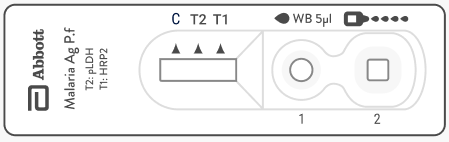 |
| RDT cassettes with two test lines to detect *P. falciparum* HRP2 and pan-pLDH | | |
| Bioline Malaria Ag P.f/Pan  (Abbott, IL USA) [41] | Bioline Malaria Pf/Pan | *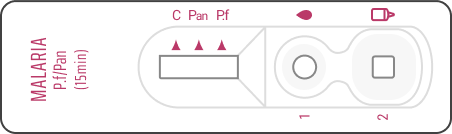* |
| First Response Malaria Ag. pLDH/HRP2 Combo Card Test (Premier Medical Corporation Ltd, Gujarat, India)[42] | First Response Malaria Pf Ag (pLDH/HRP2) | 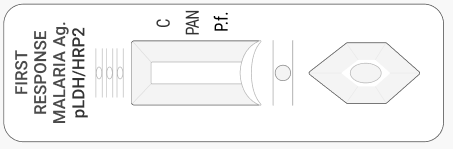 |

Supplementary Table 2. Fleiss’ kappa score calculated to measure interrater agreement on RDT results among three external panelists by country, 2023

| **Country** | **N** | **Kappa** | ***P* value** |
| --- | --- | --- | --- |
| Benin | 2200 | 1.0 | < 0.0001 |
| Côte d’Ivoire | 2200 | 1.0 | < 0.0001 |
| Nigeria | 2200 | 0.998 | < 0.0001 |
| Uganda | 3746 | 0.999 | < 0.0001 |
